# Short Versus Long Term Benefits and the Evolution of Cooperation in the Prisoner's Dilemma Game

**DOI:** 10.1371/journal.pone.0056016

**Published:** 2013-02-11

**Authors:** Markus Brede

**Affiliations:** Department of Electronics and Computer Science, University of Southampton, Southampton, Hampshire, United Kingdom; University of Maribor, Slovenia

## Abstract

In this paper I investigate the evolution of cooperation in the prisoner's dilemma when individuals change their strategies subject to performance evaluation of their neighbours over variable time horizons. In the monochrome setting, in which all agents per default share the same performance evaluation rule, weighing past events strongly dramatically enhances the prevalence of cooperators. For co-evolutionary models, in which evaluation time horizons and strategies can co-evolve, I demonstrate that cooperation naturally associates with long-term evaluation of others while defection is typically paired with very short time horizons. Moreover, considering the continuous spectrum in between enhanced and discounted weights of past performance, cooperation is optimally supported when cooperators neither give enhanced weight to past nor more recent events, but simply average payoffs. Payoff averaging is also found to emerge as the dominant strategy for cooperators in co-evolutionary models, thus proposing a natural route to the evolution of cooperation in viscous populations.

## Introduction

Altruism is a widely observed phenomenon in the social sciences, biology and economics and one might even argue that it is the fundamental characteristics that holds human society together. Its emergence and sustainability in populations of self-interested agents is conveniently modelled in the framework of evolutionary game theory [1]. Among with public goods games and the snowdrift game, the probably most widely studied example in the field is the prisoner's dilemma game, describing the simultaneous decision making of two individuals in a conflict situation, in which two options typically labelled “C” (for cooperate) and (“D”) for defect are available. Depending on the mutual choices, agents receive a payoff of 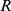 for mutual cooperation, defect against cooperate receives 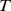 vs. 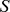 for the cooperator, and the payoff for mutual defection is 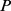. In the prisoner's dilemma these payoffs are ranked 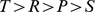 and 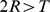, such that independent of the opponent's strategy defection always yields a higher payoff than cooperation. In contrast, mutual cooperation yields the highest combined payoff for the group. This raises the question: How can maximum group outcomes be achieved even though it is beneficial for the individual to defect?

Many previous works have addressed this question in various contexts. Nowak [2] classifies possibile solutions into five categories, amongst which are mechanisms like kin selection, group selection, direct and indirect reciprocity and network reciprocity. Starting with studies of spatial graphs [3] which have later been extended to small world [4] and scale-free networks [5] particularly the latter has found much interest in the literature in recent years, see [6]–[8] for reviews. Network reciprocity describes a “viscous” population, i.e. a situation in which individuals can only interact with a fixed set of partners and not with the whole group. Cooperation can survive to some extent, because cooperators can positively assort, thus shielding themselves from invasions of defectors. In spite of this, in the asynchronous model with probabilistic updating on spatial lattices only very limited propertions of cooperators can survive.

Cooperation through network reciprocity can be further supported by opponent selection mechanisms that enhance the shielding of clusters of cooperators [9], [10] or by including various forms of heterogeneity into the models. Some such examples of cooperation supporting heterogeneity are network heterogeneity [4], [5], [11] payoff noise [12], [13], quenched noise in payoffs [14], and various forms of unevenness in strategy pass, like learning and teaching [15], aspirations [16], [17] or others [18].

Over the last couple of years the focus in the field has increasingly shifted to co-evolutionary models, see [8] for a review. In these models an evolution of individual-specific traits at a timescale comparable to the timescale of the spread of game strategies is considered. Examples are studies on co-evolving networks [19]–[21], but more recently also investigations of co-evolving noise levels [22], aspirations [17], learning and teaching [23], [24] or co-evolving update rules [25], which have served as a major inspiration for the present paper.

Recently, Chadefaux and Helbing [26] proposed a mechanism of wealth accumulation to support cooperation. In their model wealth is created endogenously from game interactions. Agents accumulate payoffs indefinetely by playing games with stakes that are proportional to an agent's accumulated wealth. The authors report that cooperation is maximally supported when agents risk their entire wealth in every encounter whereas support for cooperation is rather low when small proportions of wealth are at stake. Risking a fixed stake of payoff in every encounter allows for an over-exponential growth of total wealth between cooperators and the study thus links wealth accumulation to (extremely) uneven endogenously created distributions of payoff and wealth.

In many ways [26] is also the starting point of this paper. I propose a model in which agents consider differently weighted accumulated (and suitably normalized) payoffs of their opponents as the basis of decisions for strategy adoption. Suitable normalization of payoffs and constant payoffs in the game exclude the mechanism of wealth heterogeneity described in [26] as the mechanism responsible for cooperation.

A weighting scheme of past and present payoff can be regarded as an agent's perspective on performance evaluation. I then proceed by considering prespectives and game strategies as co-evolutionary: agents can adopt their neighbours' game strategies (cooperate or defect), but can also adapt their perspective, i.e. the rule through which they evaluate a neighbour's success.

Specifically, I aim to answer two questions in this paper: (i) is the supporting effect of accumulation necessarily related to unevenness in wealth (or fitness)? and (ii): what is the influence of different ways to weigh past and present payoffs on cooperation? Can agent's perspectives and strategies co-evolve in such a way that cooperation is supported? After explaining the details of the model, I proceed to address the first question in the next section. Following from this, the co-evolution of strategies and perspectives is investigated and the results are summarized and discussed in the context of the literature in the concluding section.

## Methods

More specifically, I consider a set of 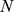 agents which are located on a spatial lattice with von Neumann neighbourhoods. Agents are engaged in a prisoner's dilemma game with their nearest spatial neighbours and can play either one of two pure strategies: cooperate (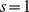) or defect (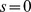). I follow a large portion of the literature and parameterize the game via
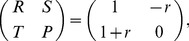
(1)thus leaving parameter 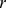 to control the dilemma strength. As usual, for 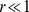 the dilemma setting is weak and for 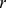 cose to one the strongest conflict between individual and group interests is found.

In every round of the evolutionary game, a randomly picked focus agent, say 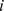, and a randomly selected neighbour, say 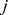, of the focus agent play a one-off prisoner's dilemma game against each of their respective neighbours. This determines their instantaneous payoffs 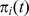 and 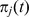. In a next step, the focus agent can adapt its strategy. This is modelled in the typical way how imitation dynamics are represented in evolutionary games, i.e. agent 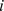 will adopt the strategy of agent 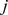 according to a probabilistic rule [27]

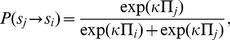
(2)where the parameter 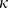 gives the noise in the strategy updating process. Thus, agents will typically adopt strategies of neighbours whose performance 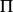 they value more highly than their own. Performance in the game is evaluated over a time horizon of the last 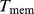 game interactions according to differently weighted current and past payoffs via
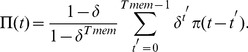
(3)In equation (3) the parameter 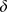 represents a discount/interest rate of past interactions. If 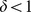 a player's evaluation time horizon is effectively shorter than 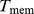 and the effect of past game outcomes on the performance measure is low. Contrariwise, 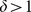 corresponds to a strongly weighted past such that performance is mainly determined by the past and the influence of the latest game interactions of a player is negligible. The limit of 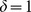 corresponds to a simple average that neither discounts nor exaggerates past events. Also note the normalization factor in Eq. (3) which ensures 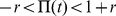, thus allowing the consistent treatment of noise in strategy propagation.

There are two straightforward interpretations of the parameter 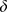. First, one can assume that the value of 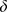 is determined externally and 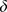 is more or less equal for all agents. In the limit of low noise values 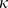 this case corresponds to a biological scenario similar to the model of wealth accumulation described in [26] (but notice the important difference that the agent's stakes in the game are independent of payoff in the present model!). Agents accumulate payoff over some time horizon given by the discount rate 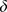 after which they can replicate. The success of their game strategies is only evaluated at this point in time. Importantly, the typical number of game interactions before payoffs are evaluated is given by the discount factor. Realistic scenarios are such with 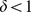 describing growth subject to depreciation, but also 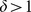 might be realistic for some organisms for which events in early life are very important to determine later fitness. The scenario of 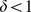 might also be interpreted as a growth process subject to decay at rate 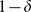, e.g.
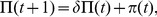
(4)and strategy spread or replication which occurs at timescales much longer than those of the growth process.

The second interpretation of 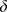 is that it reflects an agents' perspective. As such it would have to be considered as agent-specific. Agents with low 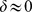 are short-termers: they will adopt strategies from others who did better than themselves in the last interaction. Agents with larger 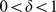 increasingly base their evaluation on past as well as on present behaviour and agents with 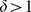 are agents that value past interactions more highly than present ones. In this setting it is natural to consider a scenario in which agents can change their perspectives as well as their game strategies. Realistically, perspective changes will occur at a slower timescale than game strategy adaptations.

In the first interpretation, to which I refer as monochrome models, the discount factor 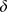 is externally set to an equal value for all agents in the game. In this setting, with a probability given by equation (3) the focus agent will only adopt the reference agent's game strategy when updating. In the second setting, that I refer to as co-evolutionary, agents will adapt their game strategies as well as their perspectives, both occuring with the probability given by Eq. (3).

In more detail, simulation experiments are carried out on lattices of varying sizes according to the following rules

- Start with a random allocation of strategies to sites such that 50% of agents are cooperators and 50% are defectors. For every agent 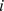 the entire payoff histories 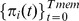 are set to the payoffs achieved in the initial population.
- In an asynchronous updating procedure, a focus agent 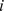 and a reference agent 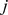 are selected and their respective current payoffs 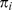 and 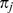 are determined and stored in their payoff histories. Only payoffs over the last 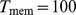 timesteps are stored and any information about past payoff beyond this is not taken into account.
- The performance measures 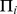 and 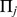 of 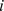 and 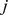 according to 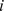
's perspective are determined. With probability 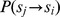 agent 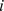 adopts the strategy (and possibly perspective) of 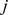.
- Iterate steps (ii) and (iii) over 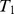 sweeps over the full lattice until a quasistationary state has been reached and then average statistics over another 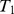 sweeps to determine the stationary number of cooperators 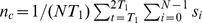.

## Results

### The case of monochrome discounting

**Figure 1:**
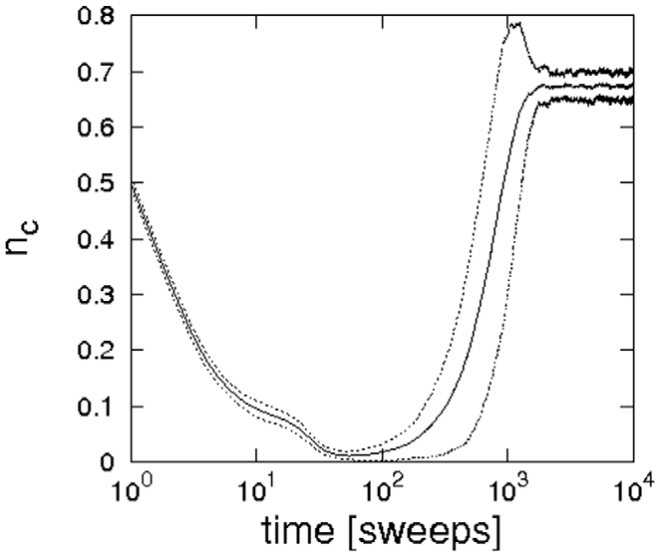
Evolution of the concentration of cooperators over time. Parameter choices are 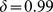, 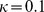, 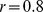 and simulations were performed on a 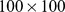 lattice. Trajectories are averaged over 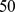 runs, dashed lines indicate two times the standard deviation.

**Figure 2:**
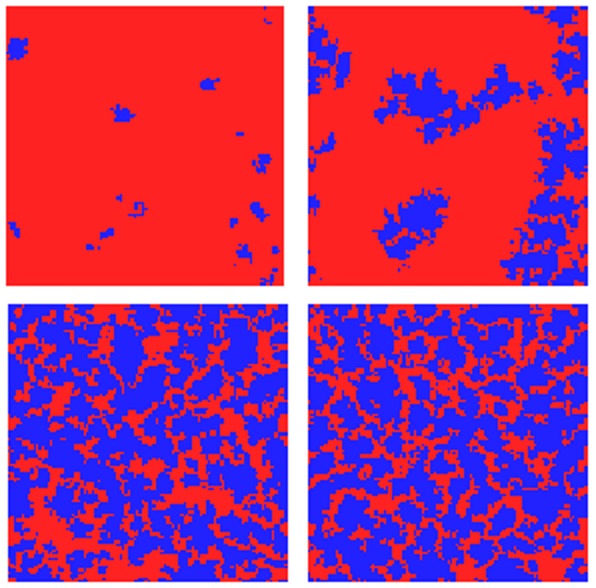
Snapshots of agent configurations on a 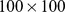
 **lattice.** The snapshots were taken after 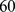, 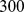, 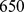, and 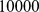 full update sweeps. Parameters are 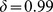, 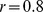 and 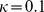.

In this section I will consider the case that all agents share the same interest/discount parameter 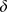. Figure 1 shows a typical trajectory for the evolution of cooperation in a tough dilemma situation (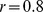) when strategy spread is influenced by memory of past payoffs (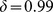). Illustrations of typical arrangements of cooperators and defectors at the various stages of the evolution are given in Fig. 2. Starting with a random allocation of cooperators and defectors, the evolution follows the known pattern: defectors can earn the highest payoffs in random arrangements and consequently they spread over a large part of the lattice, such that only little pockets of cooperators remain. Once an ordered arrangement of cooperators and defectors has been reached, clusters of cooperators may start to expand again until an equilibrium between cooperators and defectors is reached.

Crucially, however, when strategy spread is based on the immediately preceding payoff earnings, coexistence equilibria between cooperators and defectors are only possible for very weak dilemma strengths. This case corresponds to a discount rate of 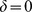 in my parameterization. For the noise level and neighbourhood specifications used in the above simulations, a second order phase transition is found at a critical value of 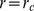. This transition belongs to the universality class of directed percolation [6], [27]. In typical simulations based on updating of recent payoffs (i.e. 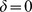) this critical dilemma strength at which cooperators go extinct is found at 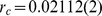 [6]. In stark contrast, the simulations illustrated in the panels of Figs 1 and 2 indicate that if updating takes account of past game outcomes, cooperation can survive for much larger dilemma strengths than expected.

**Figure 3:**
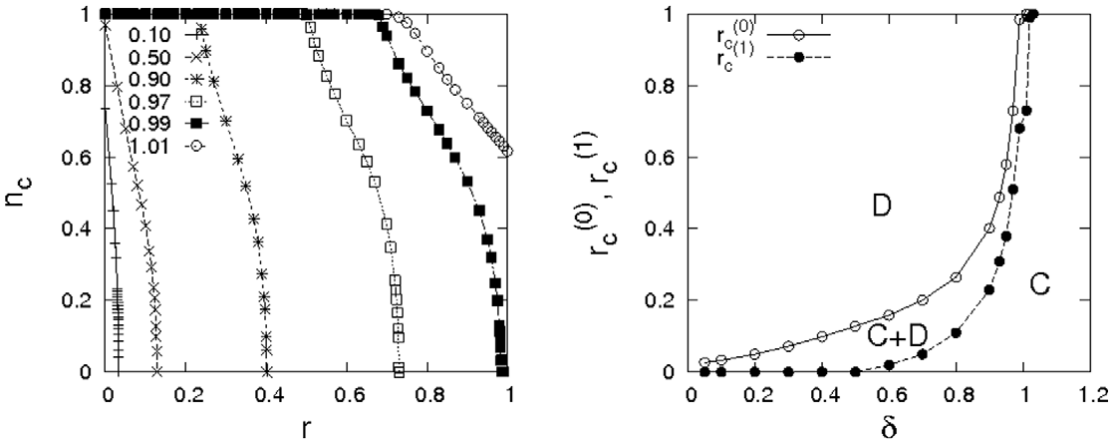
Dependence of the frequency of cooperation on the dilemma strength. (left) Dependence of 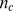 on 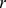 for various values of 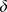 (see legend) for 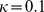 on a 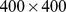 square lattice. (right) Phase diagram depicting the extinction threshold of cooperation (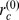) and the extinction threshold of defection (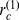) depending on the timescale of payoff evaluation 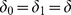 for a square lattice with 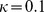. Error bars are smaller than the size of the symbols. Notice, that cooperation can always dominate if 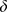 is slightly larger than one.

By presenting a more thorough investigation of phase boundaries, the panels of figure 3 reinforce this point. The data illustrate that the support for cooperation grows systematically, when more and more emphasis is placed on the evaluation of past payoffs. A more detailed analysis of the phase transitions where cooperation or defection die out is given in the bottom panel of the figure. For 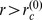 cooperation dies out and defection dominates, for 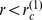 cooperation dominates and in between for 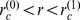 mixed equilibria of cooperators and defectors are possible (cf. the regions labelled “C”, “D”, and “C+D” in Fig. 3). The detailed analysis of the phase transitions reveals that for choices of 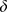 slightly greater than one, cooperation can even dominate over the entire range of dilemma strength, thus resolving the dilemma in any situation!

What is the reason for the strong support for cooperation from the evaluation of past payoff? To understand this, consider a defector at the boundary of a cluster of cooperators. In a typical configuration, such a defector exploits a number of cooperators at the cluster's boundary and thus achieves a larger payoff than most cooperators in its neighbourhood. However, discounting payoff essentially introduces a delay between the start of the exploitation of the cooperators by the defector at the boundary and the time when this exploitation becomes visible in the payoff histories. Hence, there is a period of time during which the effective performances of the cooperators at the boundary appear superior due to good past payoff results (when they were still surrounded by cooperators) and thus, even though a boundary defector may have obtained larger payoffs in the recent past, it cannot immediately invade surrounding cooperators. Clearly, this delay slows down the spread of defectors and thus promotes the spread of cooperators. The delay until most recent payoffs become effective in agent's performances is related to the parameter 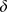. Larger 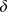 implies a longer delay. Hence, following this argument one also expects that the support for cooperation grows with 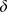 which is corroborated by simulation results, cf. Fig. 3.

If one follows the interpretation of discounting as a rate of depreciation in a growth process, the above result demonstrates a remarkably strong enhancement of network reciprocity if updating is based on a measure that combines current and past payoff. This support becomes the larger the more emphasis is put on payoff events farther in the past. However, already close to equally weighing past and current payoff can allow the dominance of cooperation over the entire range of dilemma strengths.

It is also worthwhile to emphasise that this support for cooperation is achieved without the extreme wealth heterogeneity that is fundamental to cooperation in the model of [26]. In the present model the stakes in the game are independent of past success and wealth distributions of cooperators and defectors are the same as in the typical evolutionary prisoner's dilemma game on a square lattice.

If one follows the second interpretation of discounting as a subjective performance measure of individuals, the above results immediately raises the question if perspectives that support cooperation are evolutionarily stable. In other words, can low discounting survive or even dominate in a population of self interested agents? I address this question in the next section.

### Co-evolution of cooperation and perspectives

#### Continuously varying perspectives

In this section I consider a model in which agents can inherit the perspective of the reference agent when adopting its game strategy. As before, simulations are initialized with random allocations of 50% cooperators and 50% defectors, but now agents are also assigned a perspective 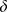 chosen from a uniform distribution 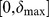. To account for mutations, in some cases perspectives are slightly modified when they are adopted. Technically, this is implemented as a 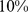 chance that a small random number from the interval 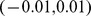 is added to the adopted perspective. Boundary conditions 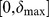 are strictly enforced in this process. Furthermore, I also consider the role of mutations when strategies are passed on between agents. In such a case, with a small rate 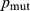, agents adopt a randomly selected game strategy and a randomly selected new perspective.

**Figure 4:**
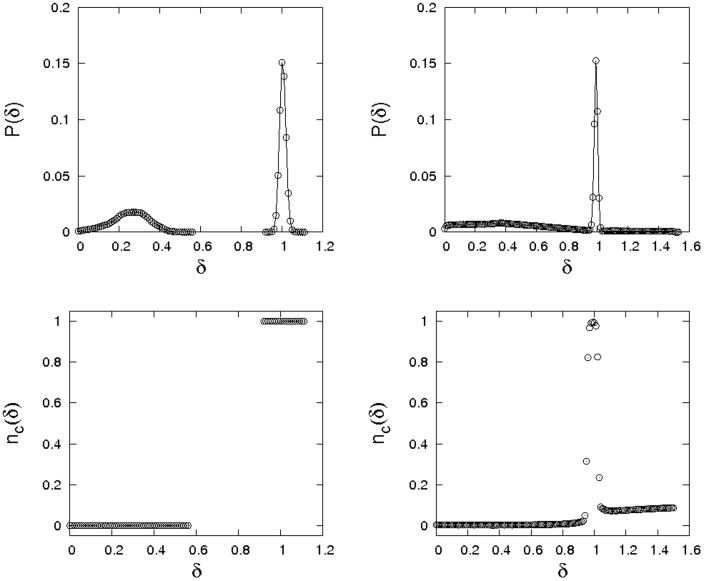
An illustration of the stationary states in the co-evolution of perspectives and strategies. The panels give distribitions of evolved perspectives (top) and the dependence of the average frequency of cooperators on perspectives (bottom). The figures show results from simulations on a 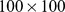 torus with 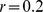, 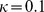. In the right hand panels a small chance (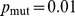) of misperception when adopting another agent's strategies is included. In case of a misperception, an agent adopts the opposite of the game strategy of the reference agent and chooses a new perspective uniformly at random from the interval 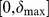. Without misperceptions around 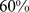 of agents are cooperators, with misperceptions only around 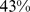 are cooperators.

By recording stationary distributions of perspectives (top) and giving statistics of the frequencies with which agents with a certain perspective are cooperators (bottom), figure 4 summarizes typical simulation outcomes on a 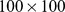 torus for situations with and without mutations. In both cases, a clear separation of agents into two groups becomes apparent. One peak of the bimodal distribution of perspectives corresponds to agents who almost always defect, the second to agents who almost always cooperate (cf. bottom panels of Fig. 4).

If mutations are included, the distribution of 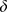-values for defectors becomes much broader and a further distinction between two classes of defectors becomes apparent. Agents with 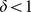 are almost always defectors, whereas agents with 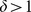 tend to be defectors, but have an around 15% chance of being a cooperator.

Further experiments for changing levels of noise in strategy propagation demonstrate that the location of the defector peak at around 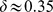 is an artifact of the level of noise in strategy propagation. In fact, for 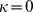 one finds a much sharper first defector peak at 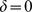. Noise in strategy propagation and neutral drift of perspectives in the large areas of the lattice occupied by defectors then allow for the survival of 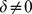 defectors.

**Figure 5:**
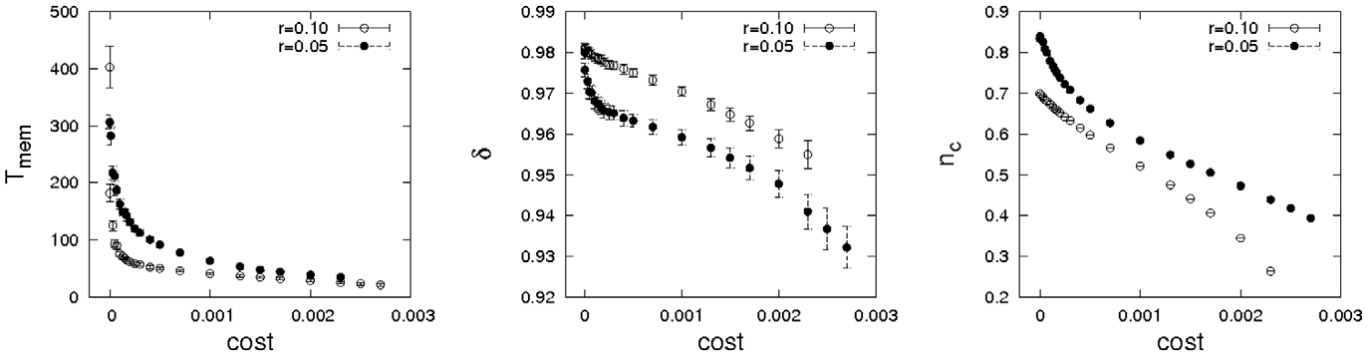
Dependence of evolved memory lengths of cooperators, discount factors of cooperators, and density of cooperators on costs per unit of memory. The setup is the same as for the previous figure, but 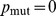. Timescales are bounded by an upper limit of 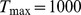, and simulations were performed on a 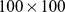 torus using 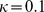.

It is instructive to investigate a further extension of the model and allow memory lengths to co-evolve with strategies and perspectives. Since defectors make little use of past information, memory lengths of defectors are subject to random drift while memory lengths of cooperators keep increasing until the marginal benefit of further increases is counteracted by the noise level in strategy propagation. In real-world situations memory is often associated with a cost. Including such a cost per unit of time in memory, memory lengths of defectors quickly converge to zero. In contrast, memory lengths of cooperators reach an equilibrium at which the costs of memory balance the advantages for strategy spread. Figure 5 illustrates data gleaned from simulation experiments with co-evolving memory lengths, perspectives, and game strategies. The first panel gives the dependence of stationary memory length of cooperators on costs, the second the corresponding stationary perspectives and the third the stationary densities of cooperators. In all shown cases a coexistence equilibrium of cooperators and defectors could be reached. This becomes impossible above some cost threshold, at which memory becomes too costly for cooperators to allow for meaningful long-term evaluation.

The above experiments give a clear indication that a consensus of perspectives in the population is not an evolutionarily stable outcome. Instead, defectors have a natural tendency to employ a short time perspective, whereas cooperators are natural long-term evaluators. Interestingly, however, the evolutionarily stable (and optimal as we will see in subsection) outcome for cooperators is not an overly strong emphasis on the past which was found to be a strong enhancer of cooperation in the monochrome case. Instead, perspectives of cooperators are stabilized at around 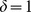 which corresponds to taking averages of past payoff.

This distinction of optimal perspectives for cooperators and defectors can be understood by a simple argument. As argued in the previous subsection, defectors who evaluate past performances when updating strategy are affected by the delay between suboptimal immediate payoffs and when they become effective in payoff histories. In their perspective, cooperators at the boundary of a cluster of cooperators appear to have larger payoffs and hence they are likely to adopt the strategy of cooperators. In contrast, defectors who only evaluate short term payoffs are unaffected. Short term defectors perceive their payoffs as larger than those of boundary cooperators and cannot easily be invaded by them. However, in the view of long term cooperators, the payoffs of short term defectors at first also appear inferior and cooperators will not immediately adopt the defect strategy. This mismatch in perspectives delays the spread of defectors and gives support to cooperation, albeit not as much as for monochrome long term evaluation in a population.

Why is averaging (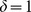) the stable strategy for cooperators? I have argued before that longer time horizons are essential for cooperators to survive. What remains to be answered is why 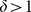 is not a stable perspective for cooperators. To understand this, note that clusters of cooperators are constantly in flux: they expand in some directions and shrink in others due to the invasion of defectors. Hence, cooperators are often surrounded by other cooperators, but might sometimes also be at the boundary and hence prone to exploitation by defectors. In case of 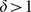, such exploitation events in the past life of a cooperator can occasionally be exaggerated, thus making the cooperator prone to adopt the strategy of a defector. In contrast, averaging (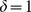) prevents the impact of chance highlights of specific events in the payoff history and only cooperators who consistently happened to be at a boundary are prone to invasion of the defect strategy.

#### Averaging in the face of short term defection: When can cooperation survive?

In the previous subsection I have demonstrated that cooperators naturally align themselves with performance measures that average over time, whereas defectors tend to evaluate payoff at a very short term basis. This underlines that (in the absence of substantial levels of noise) perspectives in co-evolutionary models will fixate at only two distinct values of 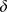. For a systematic exploration of the sustainability of cooperation in the presence of two possible perspectives I consider a discrete model. Perspectives are co-evolutionary with strategies as before, but only two perspectives, 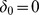 and 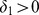 can be assumed.

**Figure 6:**
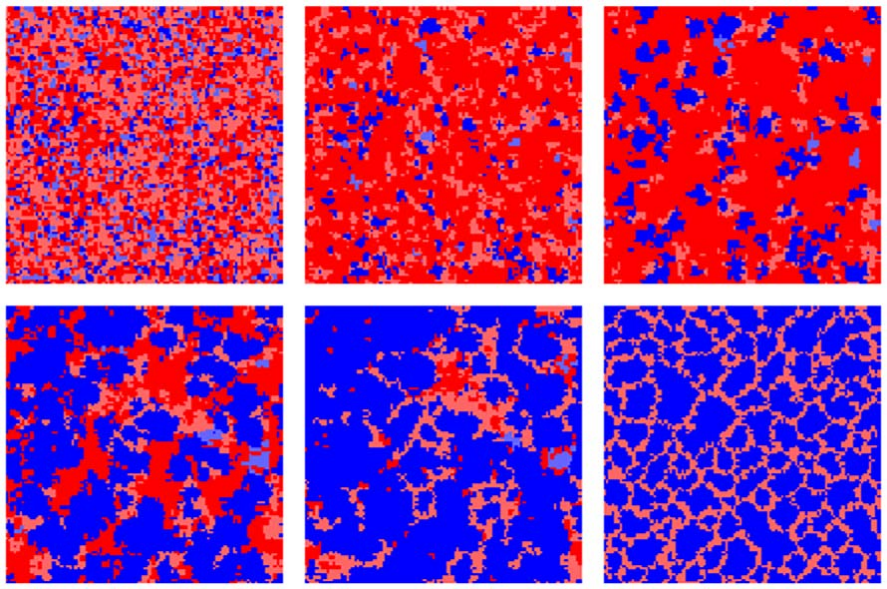
Snapshots of agent configurations illustrating the co-evolution of perspectives and strategies. The snapshots are taken after 3,20,40,70,100 and 1000 sweeps (left to right) with parameters 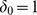 and 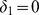. Cooperators are blue, defectors red, darkness of the colour indicates perspective, dark indicates long term (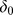) and bright indicates short term (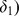. See fig. 7 for averaged trajectories.

**Figure 7:**
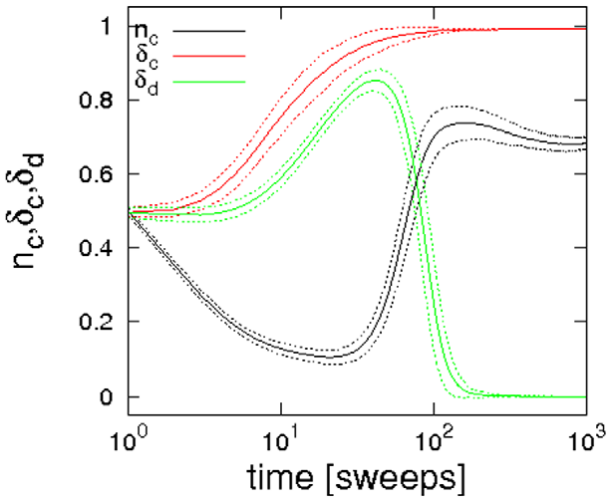
Co-evolution of perspectives and cooperation for 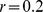
 **,** 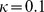 **on a** 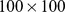 **lattice.** The lines give trajectories for the (i) average density of cooperators, (ii) average perspective of cooperators, and (ii) average perspective of defectors. Trajectories have been averaged over 100 simulation runs and the dotted lines give two times the standard deviation.

Figures 6 and 7 illustrate typical scenarios in the evolution of cooperation in the above case and several stages in the evolution can be discerned. Initially, long term horizons grow to dominance in both the cooperator and defector populations. In the first case this is the scenario described in the previous subsection. In the case of defectors, the initial long-term horizons are related to the initial payoff bonanza for defectors in random allocations of cooperators and defectors. Long time horizons dominate, because they can lock in a memory of the initially high payoffs reminiscent of the initial conditions. However, when simulation times approach the memory time horizon of 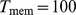, this memory starts to fade and cooperators can invade the large areas of long term time horizon defectors. Pockets of short term defectors are the only defectors that eventually survive and a fluctuating steady state pattern of long term evaluating cooperators and short term evaluating defectors is approached.

**Figure 8:**
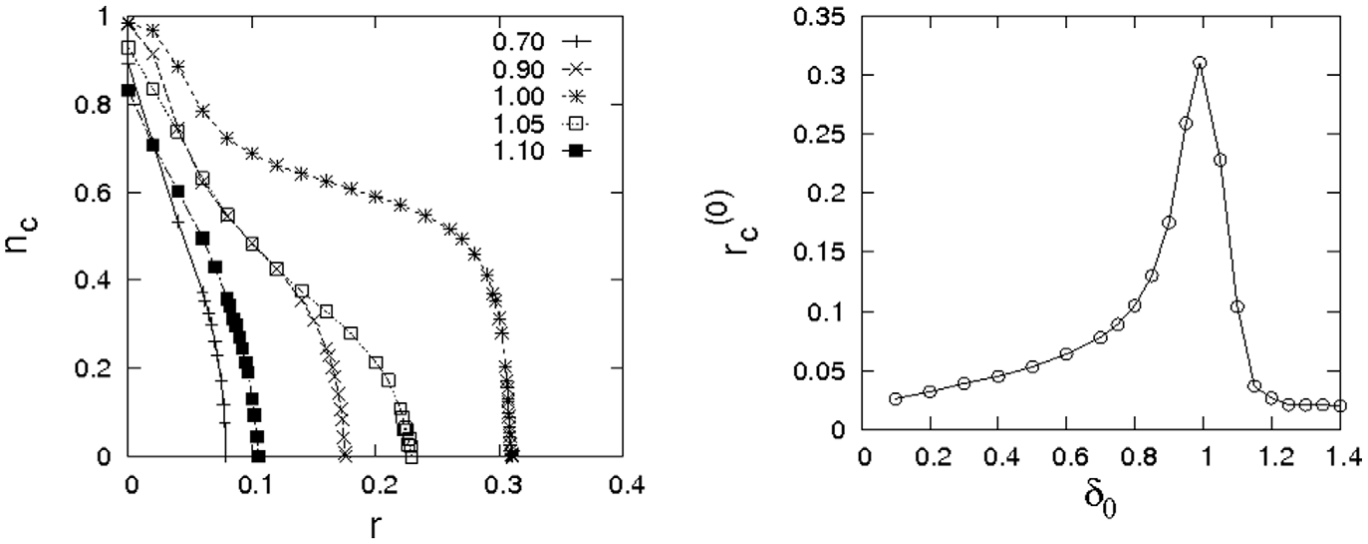
Dependence of cooperation on the dilemma strength when strategies and perspectives co-evolve. (left) Dependence of the fraction of cooperators on the dilemma strength 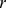 for various values of 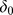 (see legend) for 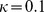 and 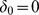 on a 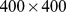 square lattice. Notice, that the optimal perspective for cooperation is no longer the largest possible value of 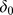 as in Fig. 3, but cooperation is maximized near 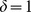. (right) Phase diagram depicting the extinction threshold of cooperation depending on the largest timescale of payoff evaluation 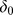 (while 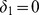) for a square lattice with 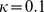. Error bars are smaller than the size of the symbols.

Figure 8 gives data for the phase boundaries between cooperation and defection for the two perspective scenario. The first panel gives the dependence of equilibrium concentrations of cooperators on the dilemma strength for various choices of 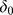. In no case can cooperators dominate the population (in contrast to the monochrome setting discussed previously), but coexistence equilibria of cooperators and defectors are still possible even for rather tough dilemma situations. The data clearly demonstrate that the support for cooperation is maximized very close to 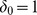, i.e. the perspective that averages payoff. This finding is reinforced by an analysis of the extinction threshold for the mixed phase given in the bottom panel of Fig. 8. The extinction thresholds 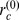 have a sharp peak around 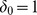. Exaggerating recent or past events in payoff histories strongly reduces support for cooperation and for 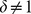 the extinction thresholds quickly approach the known phase boundary 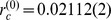 for spatial lattices with von Neumann neighbourhoods [6].

**Figure 9:**
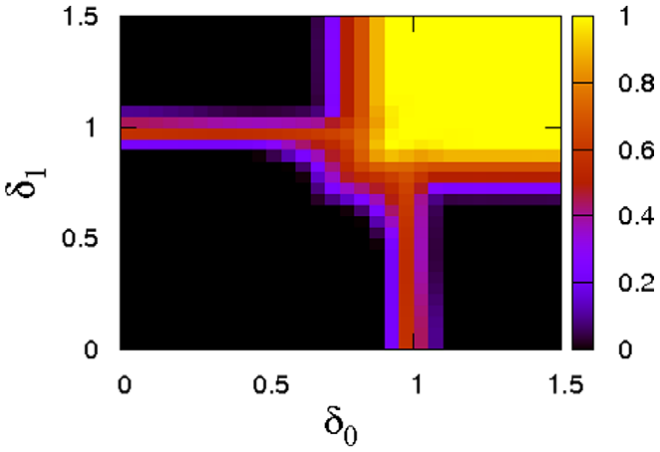
Dependence of cooperation on the choice of perspectives for 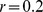
 **and** 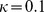 **on a** 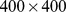 **torus.** Support for cooperation generally grows, the larger 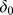 and 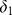, but if one of the perspectives is smaller than one, cooperation is optimally supported if the other perspective averages payoff histories (i.e. 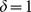).

We thus see that cooperation not only naturally associates with an ‘averaging’ perspective, but averaging is indeed also the perspective that maximizes support for cooperation. Strictly speaking, the above experiments only demonstrate this for competition between a long term perspective and basing performance on payoffs from the last game interaction (i.e. 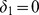). Some further experiments clarify the situation for competition between arbitrary perspectives, cf. the map plot of Fig. 9. These results clearly highlight that over a large range of 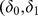) values cooperation is maximized when it can associate with averaging. In fact, this is always the case, if 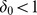 or 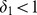. Only if 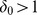 and 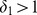 the scenario described in the previous subsection applies. In this regime, cooperation grows the larger the value of delta.

#### Coupling between strategy and perspective pass

In the previous subsection it has been assumed that game strategies and perspectives are passed on simultaneously when an agent imitates another. How important is this tight coupling between the passing on of strategy and perspective? To investigate this problem, a further modification of the original models is introduced. Now, if a focus agent decides to imitate a reference agent, game strategy and perspective are imitated probabilistically. Three situations need to be distinguished. In the first, with probability 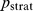 the focus agent will only copy the game strategy of its reference. In the second, with probability 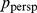 it will only copy its reference's perspective and in the remaining cases (i.e. with probability 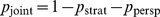) both, game strategy and perspective, are passed on. This description allows an exploration of the influence of the timescales of strategy and perspective spread as well as of the role of the coupling between strategy and perspective pass.

**Figure 10:**
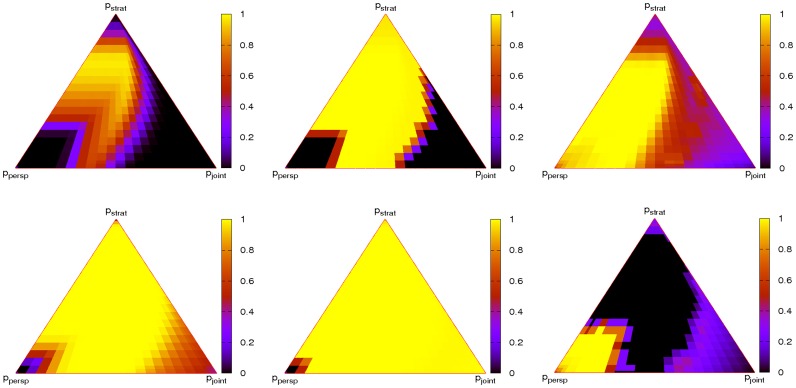
Dependence of cooperation, average perspectives of cooperators and average perspectives of defectors (left to right) on evolutionary timescales. Simulations are for 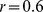, 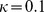 (top) on 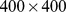 and for 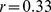 (bottom). In the plots of average perspectives of cooperators and defectors black regions indicate the absence of cooperation or defection.

Simulations have been carried out for an exhaustive exploration of the parameter space spanned by the probabilities 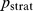, 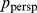, and 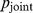. By giving the average prevalence of cooperators and average perspectives of cooperators and defectors, Figure 10 visualizes the results. Two dilemma strengths are analysed in the figure, a very tough dilemma setting with 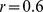 (top panels) and a lower setting with 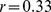 (bottom panels). Three observations stand out.

First, tight coupling between game strategy pass and perspective pass (bottom right corner) impede the spread of cooperation. In this regime defectors can associate with short term evaluation and thus – by a mismatch of perspectives as argued before – inhibit the spread of cooperators. In the case of looser coupling in strategy and perspective pass, this association becomes less strict. Whereas surviving cooperators are still always associated with long-term evaluation, increasingly more long-term evaluating defectors exist. In a scenario of monochrome long-term perspectives, however, cooperation finds much more support, see section.

Second, also very fast strategy pass does not benefit cooperation, because it slows down the spread of perspectives and thus allows the separation of typical perspectives of cooperators and defectors (see top corner in Fig. 10).

The third observation from Fig. 10 is that when rates of strategy pass are much slower than rates of perspective pass defection is favoured. To see this, recall that long-term evaluation benefits cooperation, because there is a delay between the start of the exploitation by defectors and the time when this exploitation becomes apparent in effective payoffs. During this time interval defectors are prone to invasion by cooperators, but cooperators are ‘protected’ from invasion by defectors through their payoff histories. Very slow game strategy pass reduces this advantage, since for the spread of game strategies payoff histories have an effective length of 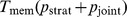. Hence the respective delay times become shorter. Incidentally, this also strongly increases the pressure towards an evolution of longer memory lengths if memory lengths are allowed to co-evolve with strategies.

## Discussion

In this paper I have investigated the impact of performance evaluation subject to variable time horizons and different discounting schemes on the evolution of cooperation in the prisoner's dilemma. I demonstrate that accounting for past success when updating strategies can strongly influence network reciprocity. Support for cooperation by network reciprocity is found to grow, the stronger past payoffs are weighted when evaluating the performance of an agent in the game. The finding helps to disentangle the effects of wealth heterogeneity and payoff accumulation described in [26]. The present model demonstrates that cooperation can be supported by an accumulation scheme without the endogenous generation of extremely uneven wealth distributions as in [26]. In some ways, one may see this as similar to the effect of a discrepancy between strategy adaptation speeds and game speeds described in [28] or the possibility that an inferior strategy with long memory can sometimes beat a superior strategy with short memory described in a somewhat different context in [29].

More importantly, I have demonstrated that low discounting is not an evolutionarily stable strategy. In co-evoluationary settings, one typically finds that defectors associate with very short term evaluation rules whereas cooperators tend to be long term evaluators. The scenario is thus in some regards similar to an essential innovation in the model of [21]: even though agents engage in the same game they have the ability to perceive this game differently.

The presence of short-term evaluating defectors destabilizes the arrangement of long-term evaluating cooperators. Due to the fluctuating nature of clusters of cooperators, very strong emphases on past payoffs are no longer the best evaluation rule for cooperators. Instead cooperation typically associates with averaging, i.e. equally weighing past and present game outcomes. Simulation experiments underline that averaging is the evolutionarily stable strategy for cooperation in the face of short term evaluating defection.

Interestingly, the averaging performance evaluation rule also maximizes the support for cooperation in co-evolutionary settings. A careful investigation of phase boundaries shows that the co-evolution of performance evaluation rules and game strategies strongly reduces the support that cooperation can gain from long-term evaluation. Consequently, phases in which cooperation dominates the entire system are no longer possible on square lattices. However, mixed equilibria between short term defectors and averaging cooperators are still possible, extending the range of dilemma strengths in which cooperation can survive well beyound the extinction thresholds of cooperation in the standard setting [6].

The described separation of performance evaluation rules for cooperators and defectors is affected by the details of strategy and performance evaluation rule updating. Tight coupling in the spread of both traits as well as timescales for game strategy spread much faster than those of the spread of the performance evaluation rule favour this separation. One might argue that the latter corresponds to socially realistic situations when individuals change their behaviour at a timescale much faster than the timescale at which they modify their underlying belief set. The separation of performance evaluation rules between defectors and cooperators vanishes, if strategies and performance rules evolve at similar timescales and are inherited independent of each other.

Even though the behavioural model investigated in this paper is extremely simple, it is tempting to speculate about the wider societal implications of the presented results. The present paper suggests that short term evaluation is a hallmark of defectors, i.e. behavioural strategies that benefit the individual at the cost of society. Inducements to base the evaluation of business leaders on long term performance have long been discussed in the media. The presented results seem to suggest that this would not only lead to a fairer society, but that it might also strengthen cooperative (i.e. group-benefitting) behaviour in society.
